# Engaging Men in Prenatal Health via eHealth: Findings From a National Survey

**DOI:** 10.2196/pediatrics.9513

**Published:** 2018-08-09

**Authors:** Michael Mackert, Marie Guadagno, Allison Lazard, Erin Donovan, Aaron Rochlen, Alexandra Garcia, Manuel José Damásio, Brittani Crook

**Affiliations:** ^1^ Center for Health Communication The University of Texas at Austin Austin, TX United States; ^2^ Department of Population Health Dell Medical School Austin, TX United States; ^3^ School of Media and Journalism University of North Carolina at Chapel Hill Chapel Hill, NC United States; ^4^ Department of Educational Psychology The University of Texas at Austin Austin, TX United States; ^5^ Departamento de Cinema e comunicação Multimédia Universidade Lusófona de Humanidades e Tecnologias Lisbon Portugal

**Keywords:** health communication, fathering, expectant fathers, prenatal health

## Abstract

**Background:**

Pregnancy outcomes in the United States rank among the worst of countries with a developed health care system. Although traditional prenatal health primarily focuses on women, promising findings have emerged in international research that suggest the potential of including men in prenatal health interventions in the United States. eHealth apps present a promising avenue to reach new and expectant fathers with crucial parenting knowledge and healthy, supportive behaviors.

**Objective:**

The aim was to explore the perceived role of men in prenatal health, acceptability of eHealth to positively engage men during pregnancy, and participant-suggested ways of improving a prenatal health app designed for new and expectant fathers.

**Methods:**

A nationally representative sample of adult males (N=962) was recruited through an online survey panel. A third-party market research and digital data collection agency managed the recruitment. The sample had a mean age of 30.2 (SD 6.3) years and included both fathers (413/962, 42.9%) and non-fathers (549/962, 57.1%). Nearly 12.0% (115/962) of participants had a partner who was pregnant at the time of the survey.

**Results:**

Despite perceived barriers, such as time constraints, financial burdens, and an unclear role, men believe it is important to be involved in pregnancy health. The majority of participants (770/944, 81.6%) found the site to contain useful and interesting information. Most substantially, more than three-quarters (738/962, 76.7%) of the sample said they would share the site with others who would benefit from the information. Participants recommended the addition of interactive modules, such as a financial planning tool and videos, to make the site stronger.

**Conclusions:**

We explored the use of targeted eHealth to introduce men to prenatal education. Results indicate men are favorable to this intervention. Additional refinement should include interactive tools to further engage men in this important issue. Reaching men at the prenatal phase is an early “teachable moment”—where new/expectant fathers are open to information on how to help their partners have a healthy pregnancy and promote the health of their unborn children. Findings will further inform best practices for engaging men in pregnancy, which is crucial for improving maternal and child health outcomes in the United States.

## Introduction

Maternal and infant mortality rates in the United States rank among the worst of all high-income countries. The most recent vital statistics reports estimate 6.15 infant deaths per 1000 live births and 15.8 to 28.0 maternal deaths per 100,000 live births—with marked disparities in outcomes among different races/ethnicities [1,2]. It is estimated that 60,000 women each year also suffer severe pregnancy-related issues considered “near-miss” maternal mortality [2]. Improving these outcomes is a goal of the US Healthy People 2020 initiative as well as a target of the United Nations Sustainable Development Goals. Health care access and social support during pregnancy can reduce risks that are known to drive maternal and infant mortality and morbidity [2,3].

To combat burdensome pregnancy outcomes, public health interventions and social marketing campaigns have played a key part in disseminating important information [4-6]. These interventions have been beneficial in promoting prenatal visits to health care providers, reducing mother-to-child transmission of diseases, and increasing breastfeeding practices [4,7,8]. Despite the success of these interventions, they have typically left male partners outside of a defined role in prenatal health care [9-12].

The inclusion of men in pregnancy-related education programs has been shown to increase provider visits during pregnancy, reduce maternal-fetus HIV transmission, and bolster postpartum best practices such as breastfeeding [13]. Research also suggests that positive male involvement during pregnancy is directly associated with positive male engagement in the formative days of a child’s life. The effect of involving men in pregnancy-related decisions has been shown to open up communication between the partners and increase the father’s efficacy in caring for the child [14].

Even though international research indicates that including men in these initiatives can improve outcomes, pregnancy continues to be a domain where men feel “invisible” and “sidelined”—prompting calls for interventions that educate and engage men on prenatal health topics [5,9,10]. Due to multiple factors, such as negative attitudes of health providers and time constraints, men are left feeling generally unwelcome in the arena of prenatal health. Research has identified several perceived barriers to men being involved in pregnancy-related health issues. These include having to work, having no time, having an unclear role, cultural norms, and the expense of such programs [10,11,15]. Given these commonly cited obstacles, the potential of using electronic health (eHealth) to educate men and motivate them to be more involved in prenatal health is encouraging [11,15]. eHealth has the ability to reach difficult-to-reach audiences, such as men, and to target information that may resonate better than existing programs designed predominantly for women.

The purpose of this study was to confirm promising pilot results on the perceived role of men in prenatal health and the potential of eHealth to reach them with information to increase their involvement in prenatal health (eg, attending prenatal visits to health care providers) [11,15]. This national survey stands to contribute to the body of research on how to encourage men to be positively involved in pregnancy to promote better health outcomes in the United States.

## Methods

### Participants, Recruitment, and Procedures

After Institutional Review Board approval, 962 adult males aged 18 to 40 years participated in the nationally representative survey. Recruitment and surveying occurred from March to May 2016. Participants were recruited from enrolled members of an invite-only research panel conducted by a third-party research firm that specializes in digital data collection. The participants had to opt-in to the study if they met survey criteria, which required participants to be adult males (aged ≥18 years). There was no upper age limit. The participants earned online credits for their participation in applicable surveys. Consent language and study contact information was provided before participants continued to the survey. All participants had to select “I consent to the protocol of this study” before viewing the content of the survey. The IP addresses were used to identify potential duplicate entries from the same user. Participation took between 10 to 30 minutes, with 94.4% (908/962) of the participants finishing within that timeframe. Prior to analysis, 64 surveys were dropped from the study because they were either blank or partially completed in less than 90 seconds. These 64 are not included in the final N=962.

The sample had a mean age of 30.2 (SD 6.3) years and included both fathers (413/962, 42.9%) and non-fathers (549/962, 57.1%). Nearly 12% (115/962) of participants had a partner who was pregnant at the time of the survey. The decision to include all men, not just those who were fathers or seeking to become fathers, was made due to the fact that half of all pregnancies in the United States are unplanned. This is intended as a population-level intervention, where all men in general can benefit from this knowledge.

Overall, 66.0% of the sample identified as white (634/962), 14.0% as African American (135/962), 9.9% as Asian (96/962), and the remainder as biracial, multiracial, or other (97/962, 10.1%). One-quarter (240/962, 24.9%) of the sample had received a high school diploma or GED, 20.9% (202/962) reported having some college experience, and more than half (510/962, 53.0%) reported having a college degree or higher. Most (814/962, 84.6%) agreed that they use the internet to look up health-related information.

### Site Development and Measures

The research was modeled after previous studies on the use of technology to engage hard-to-reach populations [16]. Participants were first asked to answer questions surrounding their perceptions of the role of men in pregnancy health, their attitudes toward prenatal health programs, their ideas on how to get men involved in pregnancy, and their use/nonuse of technology when searching for health information.

**Figure 1:**
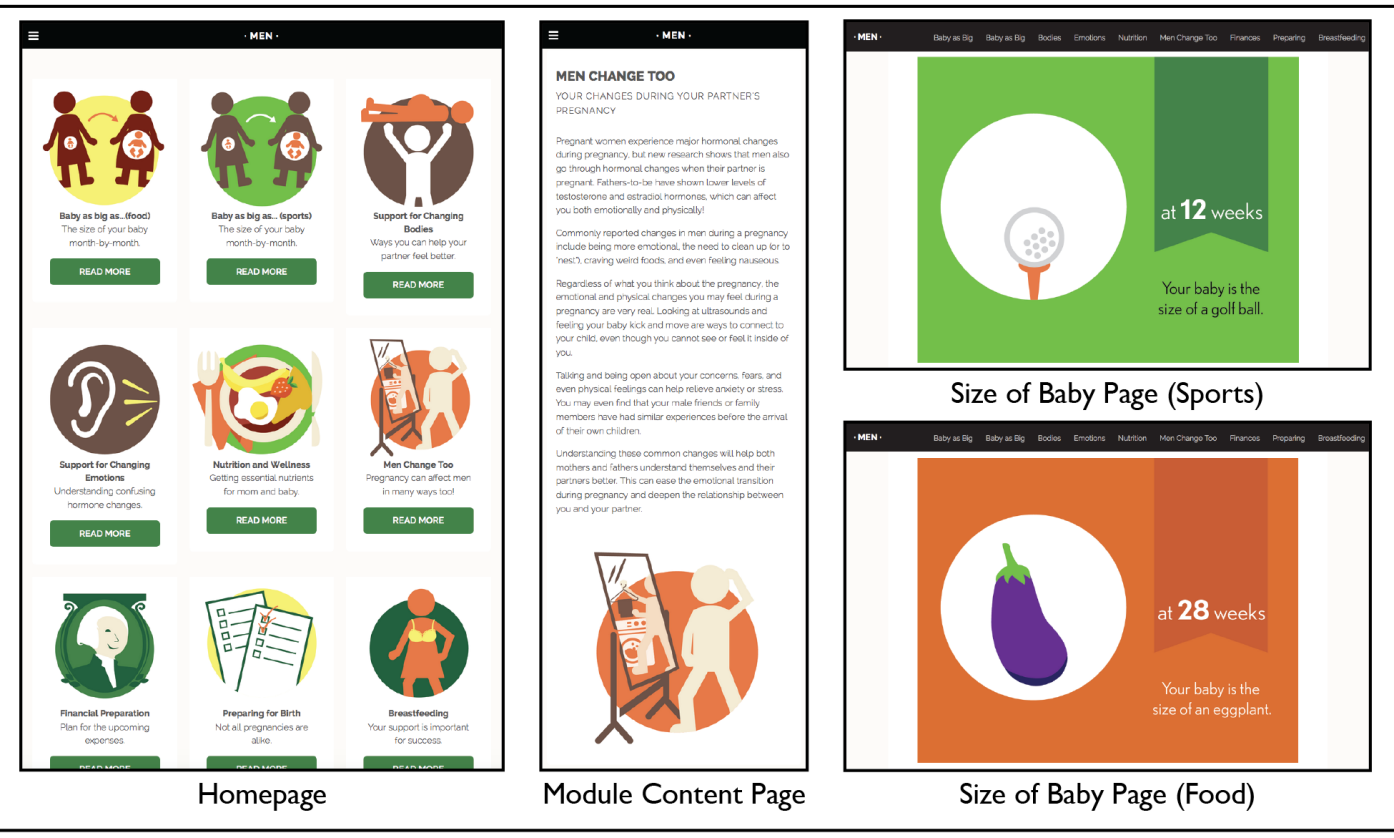
Screenshot of the Men's Pregnancy Playbook website.

Participants were then directed to navigate through the content. The site was external to the survey, allowing participants to browse and explore the content. The content of the website was written by researchers whose expertise cover perinatal health, health promotion and education, visual communication, and eHealth. The content also reflected information found in the women-focused pregnancy app, My Pregnancy Today, which included an easy-to-understand visual growth chart for fetal development.

The content of the website was intended to span across the prenatal, perinatal, and infancy phases of having a baby—from the time the parents find out about the pregnancy to approximately 1 year of age. The educational modules incorporated themes such as financial preparation, nutritional wellness, breastfeeding information, the importance of a birth plan, and how to recognize obstetric emergencies.

The website was built with a responsive design approach, which ensures content is optimally shown in the users’ environment and not platform specific. Drawing from popular social media formatting, participants could access a module’s content by clicking on icons, title, or description on the home page or from the navigation bar (see Figure 1). Guidelines from the Centers for Disease Control for ensuring clear communication—such as sufficient use of white space, consistent headings, relevant visuals, and plain language—were followed during development [17].

After exploring the website, participants were asked questions using 7-point Likert scales from 1 (strongly disagree) to 7 (strongly agree). They were also asked open-ended questions related to site content, such as what they found most useful and what could be done to make the content better. The questions were guided by previous work done on engaging men in prenatal and pregnancy health [10,15]. An adequate level of health literacy was found among the sample and was identified using three valid and reliable self-report items reported by Chew et al [18]. All content and survey questions were in English.

### Analysis

Both qualitative and quantitative methods were used to analyze the data. Descriptive statistics were used for the quantitative elements of the survey, including means, medians, and standard deviations. A mean score of greater than 5 on a 7-point scale was considered a positive validation. For the qualitative elements, a graduate student researcher and a faculty member of the research team conducted a thematic analysis by independently reviewing each written-in, open-ended answer for relevant and recurring topics.

## Results

Results are organized by the relevant themes that emerged during analysis. Results of the 7-point Likert scale items, taken both preexposure and postexposure to the app, are included in Table 1.

**Table 1:** Pre- and post-website exposure survey questions (N=962).

Item	Preexposure, mean (SD)	Postexposure, mean (SD)
It is important for a father to be involved in pregnancy health.	5.8 (1.4)	
I would attend a prenatal health class at a clinic or doctor’s office.	5.6 (1.4)	
I want to be involved in pregnancy health when the time comes.	5.7 (1.4)	
I believe knowing about pregnancy is useful.		5.8 (1.4)
It is important to know about things that could hurt your baby during pregnancy.		5.8 (1.3)
If I know about things that could hurt my unborn baby, I will try to help my partner avoid them.		5.9 (1.3)
I can take a lot of action to ensure my baby is healthy.		5.8 (1.3)
I am concerned that my baby will not be healthy.		4.9 (1.7)
Complications and unhealthiness can be life-threatening to a baby.		5.8 (1.4)
Complications and unhealthiness can be life-threatening to a woman.		5.8 (1.3)
It is hard to make sure my baby is healthy because I am not the one who is pregnant.		4.9 (1.6)
I thought this website was easy to use.		5.7 (1.3)
This website contained useful information.		5.7 (1.3)
I would use website like this to learn more about pregnancy.		5.6 (1.3)
I would prefer to have this website in an app form.		5.0 (1.6)

### Role of Men in Prenatal Health

Participants felt strongly that it is important for a father to be involved in pregnancy health (mean 5.8, SD 1.4) and that it is important to know about things that could hurt an unborn baby during pregnancy, such as second-hand smoke (mean 5.8, SD 1.3). Participants strongly agreed that if they knew about things what could hurt an unborn baby, they would do their best to help their partner avoid them during pregnancy (mean 5.9, SD 1.3). Although most agreed that it could be hard for men to be involved in pregnancy health (eg, getting mixed-messages about what partners want; 583/962, 61.0%), there was strong support for getting positively involved in pregnancy health when the time came in their own lives (mean 5.7, SD 1.4). One participant explained:

I have one child and finding information online was a big help. Whenever there were things we didn’t understand immediately we looked online then spoke to doctor to validate at next visit. But I think society in general doesn’t give fathers enough credit and makes them less important so it is easy to be discouraged to be involved...

### Visual Elements and Site Relevance

Participants were asked a series of questions regarding the overall look and feel of the site as well as how relevant the site information was to their lives. The majority of men (698/962, 72.6%) responded positively to the graphics on the site, indicating they liked the overall look of the illustrations and color scheme. Participants agreed that the layout was well structured (mean 5.57, SD 1.35) and that the site looked cohesive (mean 5.47, SD 1.34). The majority also felt that there was “just the right amount of information on the site” (684/962, 71.1%) and that they understood all the information presented (770/962, 80.0%). A participant wrote in:

I appreciated the inviting graphic design and clean, simple layout. The site was logically formatted and contained useful and easily digestible information.

### Usefulness of Content and Future Use

Participants were asked how useful and interesting they found each content module (eg, breastfeeding information) and indicated the content on financial preparation was most *useful* (756/962, 79.0%; mean 5.7, SD 1.3) followed very closely by the nutrition information (754/962, 78.4%; mean 5.6, SD 1.3). In terms of what the sample found most *interesting*, the financial preparation module was also highly rated (750/962, 78.0%; mean 5.6, SD 1.3), followed by the module on how to support a partner with changing emotions during pregnancy (732/962, 76.1%; mean 5.6, SD 1.3) and the nutrition information (740/962, 76.9%; mean 5.6, SD 1.3).

Participants generally agreed that they would use this website in the future if they were expecting a baby (698/945, 73.9%; mean 5.3, SD 1.4). The majority (717/950, 75.5%) felt that using a site such as this would improve their ability to plan and prepare for the arrival of a new baby. A respondent wrote in:

I like that this website exists at all. I have no idea how to prepare for a baby and this is the kind of website I would look for to prepare for it.

Most substantially, results indicate that more than three-quarters of the sample said they would share the site with others who would benefit from the information (738/962, 76.7%).

**Table 2:** Significant differences; fathers versus non-fathers (N=962).

Item	Fathers, mean (SD)	Non-fathers, mean (SD)	*t* test (*df*^a^)	*P* value
I think about the health of my family and friends a lot.	5.67 (1.39)	5.28 (1.46)	4.23 (946)	<.001
It is important for a father to be involved in pregnancy health.	6.02 (1.30)	5.70 (1.46)	3.51 (944)	<.001
I would attend a prenatal health class at a clinic or doctor’s office.	5.75 (1.37)	5.43 (1.50)	3.29 (946)	.001
I want to get involved in pregnancy health when the time comes.	5.92 (1.13)	5.48 (1.48)	4.84 (941)	<.001
I understand how my partner wants me to be involved in her pregnancy.	5.60 (1.34)	4.90 (1.55)	7.31 (943)	<.001
I use the internet to look up health information.	5.69 (1.37)	5.49 (1.43)	2.11 (941)	.04
This website is designed for someone like me.	5.19 (1.40)	4.86 (1.5)	3.39 (943)	.001
I can relate to the information on this site.	5.44 (1.39)	4.77 (1.51)	7.01 (942)	<.001

^a^*df*: degrees of freedom.

### Site Improvement

Participants were asked to write in ways to improve the current website and their suggestions were wide-ranging: from adding videos (“Maybe add a few how-to videos about the pregnancy process and live chat with an expert...”) to personalizing the experience by allowing users to input personal information, such as finances, to make the content more tailored. The need for dynamic and interactive information was well represented in their responses. One participant suggested:

Provide tools for the financial support: how much does a newborn cost-plug in your numbers and see how it will impact your weekly budget...Really, this is a good resource for men to get involved and take ownership of the pregnancy process, but it seems to be a bit limited on content.

Another said:

This touched the surface, but if I were interested in the financial aspects, give me links to resources to help set up savings plans, budget plans, etc.

A participant also expressed the want for an easy-to-use checklist:

I would like to see a checklist for the arrival of the baby what to buy or what to do the day when it’s time to go to hospital to have the baby.

Several participants suggested adding external links to reputable health care sites or for further reading into the topic. One stated:

There should be obvious links in each section when the user wants to learn more about a particular topic there should be (a) more detailed sources and (b) ways to access local resources near the user’s location.

The additions of a search bar to easily search for topics of interest and a forum for health care providers to answer questions were also recommended.

### Fathers Versus Non-Fathers

Independent-sample *t* tests were used to find significant differences among respondents. In particular, key differences among those who reported having children versus those who reported not having children were found. Men who indicated that their partners were currently pregnant with their first child were included in the “non-fathers” group for this analysis. Table 2 details the differences between these two groups of participants.

## Discussion

### Importance of Male Engagement

Increasing positive male involvement in pregnancy is a promising approach to improving maternal and child health outcomes in the United States. International research indicates the inclusion of fathers in prenatal education can help families recognize emergencies and better prepare for birth [5,13]. The level of male involvement during pregnancy is also directly associated with their postnatal engagement [14], which has been found to play a positive role in infant breastfeeding and sleeping best practices. The findings of this particular study answer a call for how to positively engage men in pregnancy health—moving beyond traditional interventions that only target women [19,20]. A preliminary investigation demonstrated the potential of using eHealth to engage men in this issue, and a subsequent study built on that with targeted content developed specifically for men [15,21]. The findings of this national survey confirm this is an effective way to expose men to this important content.

Recurring results show that men—even those who are not yet fathers or thinking about fatherhood—are interested in learning about pregnancy health and ways to ensure their future children are born healthy. The app is intended to be a population-level intervention to improve knowledge of healthy pregnancy behaviors across the general population. In prior studies, all adult males, regardless of sexual orientation, marital status, father status, etc, have been invited to participate. The content of this app is intended for males as partners, but there exist many forms of male involvement in pregnancy, such as being a support system for one’s family member in a multigenerational household.

Findings also indicate that men feel unsure of how to be involved and often face barriers such as time and financial constraints. Research on how best to remove these obstacles (eg, by training health care providers on how to make male partners feel welcome) is necessary to move the needle in terms of positive male engagement in pregnancy. Exploring innovative ways to communicate with this difficult-to-reach audience, such as through eHealth, are crucial.

### Future Research and Implications

The evaluation of the site was favorable and, perhaps more substantially, three-quarters of participants said they would share the site with others. This suggests a well-designed intervention could spread quickly to disseminate this kind of information. It also suggests an interest and need for content designed to help men meet recognized needs, such as emotional and financial support, during their partner’s pregnancy.

Participants’ suggestions for improvement represent the logical next steps in development: moving from a relatively small selection of static modules to interactive tools that “live” on a mobile phone as an app. Future iterations can also tailor information based on cultural, socioeconomic, and geographic factors. Social networking and sharing (eg, forums) may also be central to keeping men engaged. Men expressed their willingness to use a similar site in the future, but this study did not track participants’ actual behavior over time. Future research must link intent to behavior change in order to give a full picture of the usefulness of this type of intervention.

It is important to point out that, similar to women, men are not a monolith. Tailoring the app’s content to various audiences is critical if the content is to resonate. Expanding the app to meet the needs of Spanish-speaking men is a practical next step in development given the population growth of Latinos in the United States. This will require formative research into the cultural nuances of pregnancy that may differ from what is in the current app. By using participant feedback, the content can reach a balance between being broadly applicable and using evidence-based recommendations, but still personalized.

Finally, an app such as this can provide important data for public health researchers and practitioners. Secure data surrounding indicators such as father’s attendance at prenatal visits can be collected and analyzed to evaluate the effectiveness of the intervention. This data could inform health care providers’ ability to engage fathers during visits, explain the importance of paternal involvement in supporting prenatal health, and help everyone involved consider expectant fathers as important and active partners in supporting maternal and child health.

### Limitations

There are several limitations to this study that must be discussed. The first is that this was an online survey and participants were selected through a third-party research firm; as with most research activities, there is potential for biases in response. This survey was available only to men who had internet access and may not have captured the attitudes and beliefs of men who are not online. However, research indicates that nearly 90% of adult males in the United States are online [22]. The minority of men who are not online tend to skew older (>65 years) and are not the target demographic for this eHealth intervention.

Secondly, more than half of the sample (510/962, 53.0%) reported having at least a college degree or higher. This is significantly more than the 32% of US males who hold a college degree [23] and, therefore, the sample is not representative of all education levels. Finally, the survey was presented in English only and may not reflect the attitudes of non-English-speaking US males. The next stage of app development is to release a Spanish version. This will entail going beyond just simple English-to-Spanish translation and will require formative research with Spanish-speaking males to assess their needs as new/expectant fathers.

Although the sample was nationally representative of race/ethnic diversity, the population skewed white: with 66.0% of the sample identifying as white (634/962), 14.0% as African American (135/962), 9.9% as Asian (96/962), and the remainder as biracial, multiracial, or other (97/962). This app is designed for population-level reach, but there may be elements that may not resonate within certain racial/ethnic cultures of the United States.

Lastly, men’s perceptions of their roles and responsibilities in pregnancy health may be influenced by factors not assessed in this study. These include cultural norms and perceptions of gender roles, the relationship status of the father and mother during pregnancy, whether the pregnancy was intended or not, and more. Future research should seek to explore these influential factors and how they shape perceptions of positive involvement in pregnancy.

### Conclusions

This type of eHealth intervention, particularly once developed and delivered as a mobile app that can include ongoing interaction and data collection, could be a powerful tool for improving maternal and child health outcomes. It also serves as an example of how eHealth interventions can be effectively designed and deployed to reach nontraditional audiences, such as young men, and to address public health issues. Finally, reaching men at the prenatal phase can be considered an early “teachable moment”—where new/expectant fathers are open to information about how to help their partners have a healthy pregnancy and promote the health of their unborn children.
